# Towards Building a Distributed Virtual Flow Meter via Compressed Continual Learning

**DOI:** 10.3390/s22249878

**Published:** 2022-12-15

**Authors:** Hasan Asy’ari Arief, Peter James Thomas, Kevin Constable, Aggelos K. Katsaggelos

**Affiliations:** 1 NORCE Norwegian Research Centre AS, 5008 Bergen, Norway; 2 Department of Electrical and Computer Engineering, Northwestern University, Evanston, IL 60208, USA; 3 Equinor AS, 5254 Bergen, Norway

**Keywords:** virtual flow meter, continual learning, distributed acoustic sensing

## Abstract

A robust–accurate estimation of fluid flow is the main building block of a distributed virtual flow meter. Unfortunately, a big leap in algorithm development would be required for this objective to come to fruition, mainly due to the inability of current machine learning algorithms to make predictions outside the training data distribution. To improve predictions outside the training distribution, we explore the continual learning (CL) paradigm for accurately estimating the characteristics of fluid flow in pipelines. A significant challenge facing CL is the concept of catastrophic forgetting. In this paper, we provide a novel approach for how to address the forgetting problem via compressing the distributed sensor data to increase the capacity of the CL memory bank using a compressive learning algorithm. Through extensive experiments, we show that our approach provides around 8% accuracy improvement compared to other CL algorithms when applied to a real-world distributed sensor dataset collected from an oilfield. Noticeable accuracy improvement is also achieved when using our proposed approach with the CL benchmark datasets, achieving state-of-the-art accuracies for the CIFAR-10 dataset on blurry10 and blurry30 settings of 80.83% and 88.91%, respectively.

## 1. Introduction

The distributed virtual flow meter can provide a game-changing functionality in the oil and gas industry, and other industries requiring accurate characterization of fluid flows. With a distributed measurement capability applied to oil and gas production, it is possible to detect and locate water/gas breakthrough, monitor fractures and pressure drops as they occur, and perform a noninvasive well integrity inspection [1].

**Figure 1:**
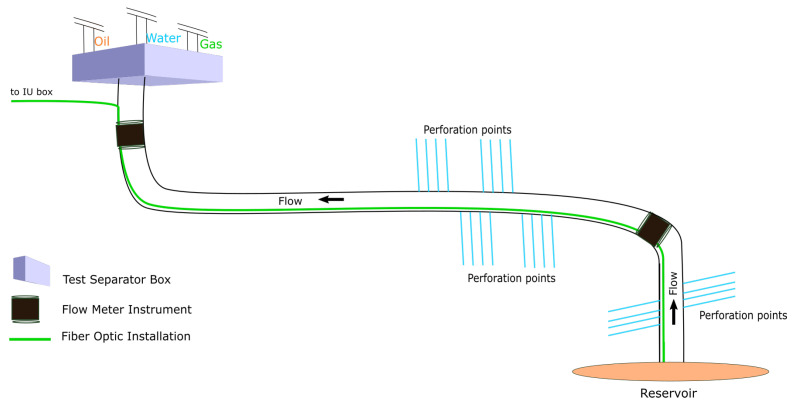
Current technologies to measure fluid content inside an oil pipeline, i.e., physical test separator, flow meter devices, and fiber optic technology. The test separator is the most accurate technology but it is intrusive and requires a redirection of the fluid flow, making it suboptimal for use in the production setting. The physical flow meter devices can provide real-time flow measurement but they are expensive, require complex installation procedures, and only provide point-based measurement. The fiber optic instrument as distributed virtual flow meter, however, can provide accurate distributed measurements with low-cost capital requirements while having the ability to provide distributed measurements. The distributed reading can help pinpoint the location of water breakthrough, leakage, and pressure drop, which can significantly increase production and reduce intervention costs of oil production environments.

Physical devices, i.e., optical flow meters, optical tomography, gamma densitometry, and test separators, can provide high-accuracy fluid flow characterization. Unfortunately, such devices are either radioactive, require an invasive operation, or do not provide distributed measurement. Figure 1 depicts an illustration of several different flow meter devices within an oil rig. The test separator works by physically separating fluid contents inside an oil pipe, and then measures the volume of water, oil, and gas individually. This type of measurement is the most accurate compared to other existing devices. However, the test separator operation requires a redirection of the fluid flow from normal operation to the separator box; thus, it is only used for getting a snapshot in time of the fluid composition. Tomography devices on the other hand, are expensive and require active reading by beaming ultrafast X-rays pulse to measure flow changes and velocity measurement [2]. Another family of flow meter devices are optical flow meters; they measure the fluid flow using the speed of sound obtained from the acoustic waves of turbulent flows. The optical flow meters have been adopted as an industry standard for cost-effective flow meter devices. However, they only provide point-based readings, and are therefore not applicable to real-time-distributed measurement. A distributed reading of the flow meter is essential because it can provide accurate information about the location of interest on where the intervention might be necessary. With this information, more effective action can be taken and the reaction time can be significantly reduced, which will translate to an increase in production well productivity within the multi-trillion-dollar oil industry. (see Figure 1).

**Figure 2:**
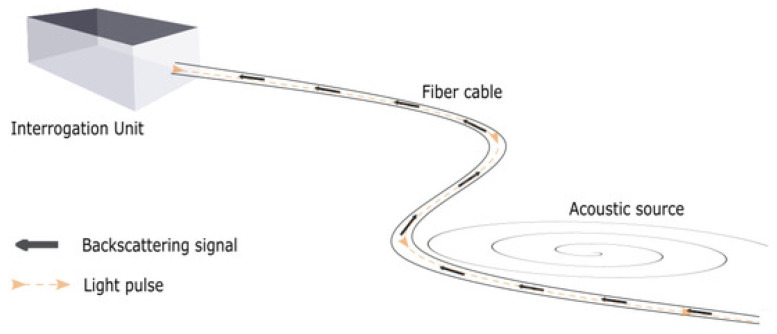
The DAS IU receiving light pulse traveling back (backscattered signal) inside the fiber cable recording acoustic signatures along the way. Source: [1].

A distributed flow meter can be defined as a metering solution that measures volume, velocity, and the fraction of fluid components in every location in the pipe. The distributed measurements can be in the form of temperature, chemical, strain, or acoustic signals. In this paper, the distributed measurements are acoustic signals acquired using a technology called distributed acoustic sensing (DAS). A DAS system consists of a fiber optic cable wrapped around (or inside) the pipe with one end of the cable attached to the interrogation unit (IU). The IU sends light pulses along the glass fibers, and interprets acoustic events around the cable via the Rayleigh backscatter mechanism; see Figure 2.

Using the acoustic signals, researchers can characterize the fluid flow inside the pipe, including the fluid volume, flow velocity, and phase-fraction of the fluids. Several techniques have been proposed to accurately estimate these quantities from the acoustic signals, including using geophysical formulations (speed of sound, Joule–Thompson coefficient, acoustic signal cross correlation, Doppler effect), and data-driven machine learning techniques from kernel function to deep learning based algorithms. The latest work from [3] shows that deep neural network (DNN) algorithms can be used to estimate the phase-fraction of the fluids from the acoustic data. Unfortunately, the accuracy of such an approach degrades rapidly when the model tries to infer from the data outside the training distribution; the situation is known as the out of distribution (OOD) problem. Unfortunately, OOD situations are common for real-world applications of modeling the DAS data.

In this paper, we explore the potential of using a different machine learning paradigm that can work well with the OOD problem. By addressing the OOD problem in machine learning, we can provide a reliable way of deploying machine-learning-based distributed virtual flow meter applications that can benefit the relevant industries as a whole. The results will bring us one step closer toward building a reliable distributed virtual flow meter. Instead of using a fixed model for inference, our proposal uses a technique called continual learning, or CL [4].

In the CL approach, the model is trained in a continuous manner; therefore, the model is always being adapted as unfamiliar fluid dynamic situations are encountered. The CL model never stops being trained, and newer data paired with their labels are continuously fed to update the model parameters, even if the new data come from OOD events. For a real-world implementation, the CL approach is suitable as a building block of a distributed flow meter, because pointwise labels characterizing the fluid flow events are often available during oil production thanks to a test separator instrument. The labels the separator provides can be used to update the trained model in a continuous manner.

Unfortunately, the CL approach has shortcomings—the most critical one being catastrophic forgetting. The forgetting problem occurs when the updated CL model learns new tasks, but forgets the previously learned tasks, therefore limiting the range of learned situations for which the model inference can provide accurate estimations. Several algorithms have been proposed to address the catastrophic forgetting problem, for example, by enhancing regularization [5,6,7], by using parameter isolation [8,9,10], and by rehearsing samples from a memory bank [11,12,13]. In this paper, we explore the last approach by using memory rehearsal to address the forgetting problem. This approach not only provides high accuracy in the CL benchmarks, but also provides seamless adaptation for noncommon data type, including DAS data.

Several CL-based algorithms to model the DAS data for distributed fluid flow estimation are explored in this paper, including GDumb [12], Riemannian Walk [11], iCaRL [14], BiC [15], and Rainbow Memory [13]. A fixed memory size is used in the experiments, so that the efficacy of each algorithm on selecting training samples and on managing memory usage can be fairly compared. Unfortunately, given the complexity and the size of the DAS data, most of the algorithms failed to provide satisfactory results.

To address the data size and memory problem, we present and test a novel sample compression algorithm that allows for the rehearsal training to store representative data within the fixed size storage. The algorithm uses the inverse cutout technique to compress and isolate the most relevant parts of the input data, and use them for rehearsal. Instead of storing the full size of input data, our proposal only stores relevant parts of the input data. Therefore, it significantly reduces the required amount of memory to store rehearsal samples, increasing the amount of rehearsal samples within the fixed size memory in the process.

We highlight that the proposed algorithm will help researchers to reduce the amount of data needed for training CL models. This is important because by reducing the amount of data per sample for rehearsal, researchers can increase the number of samples for rehearsing the CL model, which in turn corresponds to a significant accuracy improvement for estimating fluid flow in a pipe. We also provide a novel data augmentation technique for increasing the number of augmented samples for modeling the DAS data. This algorithm will help researchers to build a better model through robust augmentation.

Our contributions in this paper are the following:

- We develop a novel compressed learning algorithm based on the cutout technique to search and isolate the most relevant parts of the input data applicable to memory rehearsal.
- We provide a simple and intuitive augmentation technique that enriches image representation for non-natural image datasets.
- We perform extensive experiments on a real-world dataset to build a distributed virtual flow meter based on DAS and machine learning CL paradigm.
- We show that our proposal can also be used for natural image datasets by achieving state-of-the-art accuracies on the CL benchmark datasets.

## 2. Background

Suppose we have streaming DAS data (x, y) coming from an oil-production well, where *x* denotes the input data in the form of raw or transformed DAS data, and *y* denotes the fluid flow measurement from the well separator; *y* can be in the form of measurements for oil volume, gas volume, water volume, or combinations of them. Estimating *y* given *x* is a multihead regression task, but due to the limited research on continual learning in regression tasks, the estimation here is structured as a classification task using the bin-based regression approach with a number of classes, *C*.

### 2.1. Continual Learning

For a classification task, the deep neural network (DNN) model minimizes the empirical error by measuring the difference between the class estimation likelihood with the actual class value, and it assigns a higher penalty when the model estimates the wrong class with high likelihood. The cross-entropy (CE) loss function is used to measure the DNN classification error, and is defined as:

(1) $$L=-\sum_{i}^{C}y_{i}\log\left(s_{i}\right),$$

where *C* denotes the number of classes, $s_{i}$ denotes the likelihood-score of class *i*, while $y_{i}$ denotes the correct label for class *i*. Both the DNN model and CL-based DNN model are trained using the CE loss. The main difference between the two is the way the accuracy is measured for each model. The accuracies for the DNN model are measured using the mean-class accuracy and the mean-total accuracy defined at [16], while the CL model accuracies are measured using the highest accuracy from the final task and the average degradation of accuracies over multiple tasks; see [17]. Note that the CL model is trained on a time-filtered dataset. This setting tries to imitate real-world streaming data where each time-filtered set represents a different class distribution (in-time) from the other sets.

In this paper, the time-filtered set is referred to as task (*t*), where each task consists of several classes. The task can be structured as a disjoint task, where the classes in each task are disjoint with the other classes in the other tasks. This setup is referred to as a disjoint CL. To represent a more realistic real-world phenomenon, the CL approach can also be structured as a blurry CL, where the same class can exist in two or more tasks.

It is worth noting that both blurry or disjoint CL can be trained in an online or offline fashion. Online training means that the CL model only reads the streaming data once, while the offline training reads the streaming data multiple times until it reaches satisfactory accuracy. Furthermore, both the online and offline training can have a memory bank. The memory bank stores the samples of selected streaming data. When needed, the stored data can be rehearsed to update the CL model to avoid catastrophic forgetting. In this paper, we focus on the offline-blurry CL setting because it has similar characteristics with the real-world implementation of the oil-production environment. We refer the reader to [13] for an in-depth explanation of different CL settings.

Catastrophic forgetting occurs when the CL model learns new tasks but forgets the previously learned tasks. This is a common phenomenon in the CL training due to the heavy reliance on the iterative learning process in many machine learning algorithms, including the DNN-based algorithms. The iterative process uses incremental updates based on the new data for updating its trainable parameters. Therefore, the longer the model trains on the new data, the larger the bias towards the new tasks. The CL approaches can be divided into three categories based on the way the algorithms address the forgetting problem. They are based on (1) regularization, (2) parameter isolation strategy, and (3) memory management. The regularization approaches control the network parameter updates to mitigate the forgetting, either by introducing additional loss penalties or constraining the parameter updates by changing the parameter gradients during optimization. On the other hand, the parameter isolation strategy limits the parameter updates only to a selected range of useful parameters while keeping the other parameter unchanged. Another type of parameter isolation strategy is by adding new parameters to the existing architecture to increase the network’s ability to learn new information without forgetting the old information. Finally, the third approach is by using a memory bank and rehearsal procedure. As the term suggests, this approach stores the old streaming data to a temporary memory, and when the model is introduced to new data, the old data are also rehearsed either together with the new data or subsequently during optimization.

### 2.2. Memory-Based Continual Learning

The general framework of the memory-based CL algorithm adapted from [4] is shown in Algorithm 1. The algorithm relies on two main functionalities, namely, a sampling procedure and a rehearsal process. The sampling procedure allows the algorithm to selectively choose which data to store in the limited space of the memory bank, while the rehearsal process controls how the old data are being used to update the existing model. Several algorithms based on the rehearsal and memory approaches have been proposed to address the catastrophic forgetting problem, including Greedy Sampler and Dumb Learner (GDumb) [12], Bias Correction method (BiC) [15], and Rainbow Memory (RM) [13].
**Algorithm 1** General steps of memory-based CL.**Input**: DAS data *x*, Label *y*, Parameters θ; **Initialize**: Memory *M*← {} * *M*; Batch size *b*;
1:*x_M_*, *y_M_*←MemoryRetrieval(*M*,b)2:X,Y←*x* ∪ $x_{M}$, *y* ∪ $y_{M}$3:θ←ModelUpdate(X,Y,θ)4:*M*←MemoryUpdate(*M*,x,y)


The GDumb approach focuses on a sampling procedure according to which the spread of class distribution in the memory bank is balanced. A full retraining with the updated data before performing inference is then performed. This approach is simple and intuitive, and has shown considerable performance gains in the CL benchmark. The BiC approach introduces an additional final layer into the DNN model for calibrating the training bias. Finally, the RM approach proposes the use of Monte Carlo (MC)-based uncertainty measurements to better select which samples to store in the memory bank, and provides an extensive data augmentation (DA) proposal to enrich the replayed results.

### 2.3. Evaluation Metrics

The CL model is evaluated by using several metrics. In this paper, we use the common CL metrics defined in [17], including final accuracy ($ACC_{T}$) for measuring the model accuracy on classifying the streaming data, backward transfer ($BWT_{T}$) for measuring the catastrophic forgetting, and intransigence ($I_{T}$) for measuring the gap accuracy between offline and CL training.

$ACC_{T}$ is defined as the average reporting accuracy after the *T* tasks have been trained; thus, it evaluates the final model accuracy after all classes have been exposed to the model. $BWT_{T}$ measures the effect of learning additional tasks on the predictive capability of a model on the previous tasks. A large negative $BWT_{T}$ is a sign of catastrophic forgetting. $I_{T}$, on the other hand, measures how much, on average, the accuracy of each task differs compared to the upper-bound accuracy of a model from the non-CL setting. Following the formal definition of the CL metrics at [17], denoting by $R\in R^{T\times T}$ of the matrix of task accuracies, the $ACC_{T}$ and $BWT_{T}$ metrics are defined as:

(2) $$ACC_{T}=\frac{1}{T}\sum_{i=1}^{T}R_{T,i},.$$

(3) $$BWT_{T}=\frac{1}{T-1}\sum_{i=1}^{T-1}R_{T,i}-R_{i,i},$$

where the $R_{i,j}$ denotes the intermediate accuracy of a model on task $t_{j}$ after observing all samples from task $t_{i}$. Finally, with $ACC_{joint}$ denoting the upper-bound accuracy from the standard offline training (non-CL setting), $I_{T}$ is defined as:

(4) $$I_{T}=ACC_{joint}-ACC_{T}.$$

## 3. Compressed Continual Learning

The CL memory algorithms optimize the process of data selection (for memory storage) while also providing effective rehearsal methods. Those algorithms provide a competitive result for modeling the image data, including the MNIST and CIFAR dataset. Unfortunately, the DAS data are significantly different from the image data in terms of size and complexity. The size of the DAS data is several orders of magnitude higher than the image data and the data themselves have a lot of redundancy while containing a considerable amount of noise [1]. In the next section, we elaborate on why the existing memory-rehearsal algorithms would not work on the DAS data, and then explain how our proposal addresses the memory issues. Finally, we show how to train compressed DAS data using our proposed algorithm.

### 3.1. Rethinking Rehearsal on DAS

Rehearsal memory aims to allow the machine learning model to remember previously learned tasks by storing and retraining representative datasets with a fairly balanced distribution from the memory bank. This approach assumes that the data size is small enough, therefore enough representations can be stored within the available memory. The large size of DAS datasets presents a challenge. A total of 10 s of DAS data representing one data point requires (around) 1.2 GB of memory, while a spectrogram calculated using DAS data requires around 120 MB of memory. For bin-based regression with 72 classes, the memory requirement for storing at least 10 representations per class is extensive. As the complexity of data representation (i.e., the changes in temperature, pressure, humidity, presence of noise, and others) increases, the number of representations in the memory bank required to overcome forgetting increases dramatically. With the current state of memory-based CL algorithms, storing large amounts of data with enough representations in the memory bank is not feasible. This is not only due to the limited amount of memory but also due to the limitations in the augmentation strategies available for non-natural-image-type datasets.

### 3.2. Multistep Cutout Augmentation

It was pointed out in [13] that DA plays a crucial role in memory rehearsal because it can significantly increase the amount of sampled data through augmentation. The RM algorithm enjoys a significant accuracy improvement from extensive implementation of the DA strategy. Unfortunately, most of the DA techniques for natural images are not applicable for the DAS data because those techniques assume that the image data are invariant under translation, color inversion, rotation, and even data blending operations. Unfortunately, the DAS data are not invariant under these operations.

**Figure 3:**
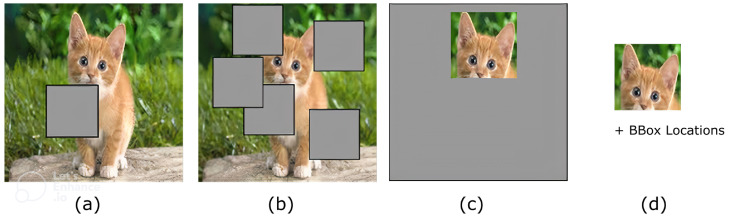
Cutout-based algorithms, including (**a**) vanilla cutout, (**b**) multistep cutout with n=5, (**c**) inverse cutout, and (**d**) compressed output of the inverse cutout for the compressed learning algorithm.

Here, we use a simple but intuitive DA strategy that can be applied to non-natural image data, such as DAS data. The algorithm is called multistep cutout; a more extensive version of the cutout algorithm introduced in [18]. The cutout algorithm uses a fixed size window randomly placed on the input data to remove (zeroing) the overlapping area between the window and the input data, and uses the modified data as the augmentation output. The multistep cutout, on the other hand, moves the cutout window multiple times (n_cut times) on the input data randomly, while zeroing the overlapping location each time the window moves. For the rest of the paper, the number of cut windows used in this augmentation technique is denoted as n_cut. Figure 3 depicts the difference between the cutout and multistep cutout outputs.

The motivation behind the multistep cutout is to teach the model to utilize the minimum (but) relevant parts of the input data responsible for making the decision on alleviating most of the noise in the input data, therefore it can generalize better. This approach is simple and applicable to non-natural image data for augmentation because the algorithm does not change the structure within the data itself.

### 3.3. Cutout Compressed Learning

One solution to addressing the data size problem is by compressing the input data. A classical *jpeg* compression is used by [19] to allow more data in the memory bank, and it has shown an improvement in CL performance. Unfortunately, this image compression technique is not suitable to compress the DAS data due to the nature of the data itself. In this section, we provide a novel approach for how we can compress the DAS data while preserving its ability to be used for rehearsal. We henceforth refer to this approach as cutout compressed learning (Cutout-CL).

The Cutout-CL algorithm works by finding the most relevant part of the input data for performing classification, and only stores that part for memory rehearsal. The most relevant part is defined as the part that occupies the smallest area of the input data but gives the lowest likelihood error (from Equation (1)). The multistep cutout algorithm is used for training the CL model to enhance augmentation as well as for maximizing the utilization of the most relevant part of the input data. An inverse cutout algorithm is (then) used for finding the most relevant part of the data. Instead of zeroing the overlapping window as in the cutout algorithm, the inverse cutout leaves the overlapping part unchanged and zeroes all the other nonoverlapping parts of the input data. The inverse cutout uses one parameter (*p*), defined as the percentage of data to leave out for compression. This approach is not only efficient for isolating important parts of the input data for sampled compression by only storing the leave-out part, but also provides fast indexing for recovery by only storing the isolated part and the bounding box location of the isolated part. By utilizing the compressed samples, the memory size in our proposal can be increased by $p^{-1}$; therefore, the new memory size $K_{n}$ is defined as $K_{n}$ = *K* × $p^{-1}$, where *K* denotes the initial memory size.

**Figure 4:**
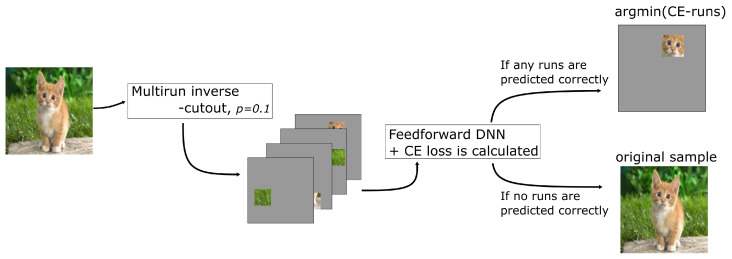
The Cutout-CL pipeline for generating compressed samples and selecting noncompressible samples.

The proposed compression technique uses both the multistep cutout and inverse cutout algorithms, and is implemented as prescribed in the following. First, the model is trained using the multistep cutout augmentation; for natural image datasets, the first step is optional due to the abundance of DA techniques for natural images. Second, at the end of each task, multiple runs of the inverse cutout algorithm are applied for each sample; here, the CE score for each run is also calculated. For each sample run with correct classification, the run with minimum CE score is used to update the corresponding sample in the sample selection, replacing the noncompressed version of the sample. It should be noted that samples without correct classification in the multiple runs, denoted as noncompressed samples, are given low priority for the episodic memory selection. Figure 4 depicts the implementations of the Cutout-CL pipeline for selecting and generating compressed and noncompressed samples. For the episodic memory selection, the compressed samples are selected randomly based on the number of maximum number of samples per class ($n_{c}$). It is important to mention that the number of samples per class needs to be balanced to avoid imbalanced class prediction. The memory selection for each class is shown in Algorithm 2.
**Algorithm 2** Compressed learning memory selection.**Input**: Compressed samples X1, Non-Compressed samples X2, Number of samples per class $N_{t}$, Compression rate *p*, Total number of seen classes $C_{t}$; **Initialize**: Memory *M*← {} * *M*;
1:**for**<c=0,1,2,..$C_{t}$>**do**                                                       ▹ *Iterate through all seen classes.*2:    *n_c_*,*X*1*_c_*,*X*2*_c_* ←*N_t_*[*c*],*X*1[*c*],*X*2[*c*]     ▹ *Populate data from the compressed learning process.*3:    $M\leftarrow M\;+\;\mathrm{RandSelect}(X1_{c},\;\min$(*n_c_*, *len*(*X*1*_c_*)))     ▹ *Random sampling through the Compressed samples.*4:    *n_c_*$\leftarrow (n_{c}-\min$(*n_c_*, *len*(*X*1*_c_*))) ∗ p▹* Update the maximum number of available spaces for the Non-Compressed samples.*5:    $M\leftarrow M\;+\;\mathrm{RandSelect}(X2_{c},\;\min$(*n_c_*, *len*(*X*2*_c_*)))         ▹ *Random sampling through the Non-Compressed samples.*


### 3.4. Understanding Cutout-CL

At the high level, our proposal works similarly to the random selection episodic memory algorithm [13]. The main difference is that the Cutout-CL benefits from the extra space the compressed data provide. It is well established that an increase in the memory space for CL learning leads to an increase in final accuracy and better handling of the forgetting problem. Moreover, it was also pointed out in [20], as well as in the attention-based DNN [21], that a machine learning model builds its feature representation by highlighting the specific part of the input data and tends to ignore the whole space of the input data. Our proposal capitalizes on this knowledge by providing a simple but intuitive compression technique that provides more sample spaces in the fixed memory bank by isolating only the relevant part of the input data. Therefore, we hypothesize that “by only storing relevant parts of the data and using them for retraining a model, we can acquire a similar (or even better) model than a model trained using the full size of input data”.

**Figure 5:**
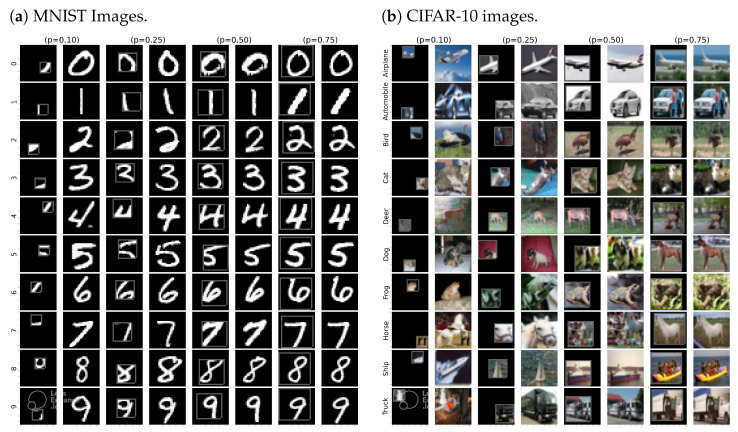
The Cutout-CL outputs where the leave-out percentage is defined by *p*. The rows show side-by-side depiction of the compressed and original images, while the columns represent different classes.

The compression rate in our proposal is controlled by *p*. A small *p* yields a larger pool of memory but it comes with a cost that the small *p* often cannot isolate all the relevant details in the images. Figure 5 shows the behavior of compressed learning output towards different values of *p* on different type of datasets. Optimizing the value of *p* requires understanding of the nature of each class in the dataset. For simplicity, in this paper, we use a fixed value *p* for all classes, and show that even with a fixed *p* and random selection of samples, a reasonable accuracy improvement can be achieved over the other CL algorithms.

## 4. Experiments

To validate our hypothesis, we turn to the empirical evidence of the Cutout-CL. In Section 4.1, we introduce the real-field DAS dataset and preprocessing step we used for the data. In Section 4.2, we show how our proposal performs on modeling the DAS dataset, and we also ablate different settings of our proposal to understand the effect of such settings in the final accuracy. In Section 4.3, we show that the proposal also provides a better model compared to the other CL algorithms for benchmark datasets, including MNIST and CIFAR-10 datasets. Lastly, in Section 4.4, we discuss some limitations of our proposed algorithms and the plans for improvement in the future.

### 4.1. Data Source and Preparations

The DAS dataset we used in this paper was from two-phase gas production with varying gas volume fractions acquired in an offshore production well. The reference data (oil and gas volumes) were acquired using a test separator instrument located on the platform topside. For the CL setup, we used bin-based regression from the combination of different range of values for both oil and gas, resulting in 72 classes.

To simplify the training process, the DAS data were transformed to stacks of spectrogram images of size (256, 486, 576) with the first, second, and third dimensions representing spatial, frequency, and time domains, respectively. Each spectrogram contains 10 s of DAS recording at 10 KHz sampling rate, and 256 spatial samples at around 1 m sampling rate. A 50% overlapping window both in time and space was used to enrich the preprocessing output. For the CL setup, the 72 classes were spread out into eight tasks randomly using blurry10 setup. We refer the reader to [13] for details on how the blurry setup is performed.

### 4.2. Continual Learning on DAS Data

In this paper, we used a ResNet18 architecture [16] to train the spectrogram DAS data. The main objective is to find out how well our proposed approach performs on the CL setting. Unless otherwise mentioned, we used an initial learning rate of 0.001, memory size of 1000, batch size of 6, cutout size of 25% of initial input data, and maximum 256 training epochs per tasks. To conserve training time for each task, the training was moved to the next task after there was no training accuracy improvement over four consecutive epochs. Additionally, due to the slow training time, only 10% of uniformly sampled DAS data were used for the experiment. Training eight CL tasks with over 10% of DAS data required (on average) more than 1 week on a single 16 GB NVIDIA P100.

**Augmentation results.** We started the experiments by measuring the upper bound or joint accuracy ($ACC_{joint}$) on the DAS data. Table 1a shows the results of the joint accuracy on several different n_cut settings of the multistep cutout augmentation. The result confirms the usability of such augmentation algorithm on the spectrogram data by providing around 5–8% of the overall accuracy improvement. We also performed the same augmentation technique on the CL setting using a random search for sample selections; the result is presented in Table 1b. Even though the improvement is smaller compared to the offline setting, the proposed augmentation performs well on the CL setting with n_cut = 20, providing the highest $ACC_{8}$ and $BWT_{8}$ of 29.73% and −39.51%, respectively. It is worth mentioning that n_cut = 1 achieves the lowest $I_{8}$ due to the low $ACC_{join}$ n_cut = 1 setting.

**Cutout-CL results.** Following the results on augmentation, we then performed Cutout-CL on multiple compression rates from p=0.05 to p=0.50. We used n_cut = 20 of multistep augmentation as the augmentation technique, and report the results in Table 2. Additionally, we also performed Cutout-CL with p=0.25 without multi-step augmentation as a comparison with other CL algorithms. The results in Table 2 confirm our hypothesis that we can generate a quality model by combining the multi-step augmentation with cutout compression to perform CL learning. Our proposal with p=0.05 achieves the highest accuracy of 32.10, which is only 15.98 points different from the offline accuracy of 48.08 from Table 1a. It is interesting to note that, considering our baseline accuracy from n_cut = 1 of 24.11, the increase in overall improvement is around 8%.

### 4.3. Continual Learning on Other Datasets

In order to compare our proposal with other CL algorithms, we also experimented with CL benchmark datasets, such as the MNIST and CIFAR-10 datasets. We follow the data preparation and hyperparameter settings in [13]; additional settings include K = 500 trained on disjoint, blurry10, and blurry30 settings with Cutmix [22] and AutoAug [23] as the DA techniques; the results are presented in Table 3. Our proposal with p=0.1 achieves state-of-the-art accuracies both for blurry10 and blurry30 of 80.83 ± 0.53 and 88.91 ± 0.20, respectively. It is worth noting that the results from other algorithms in Table 3 were populated from [13], which uses the highest test accuracies from the final task as the reporting accuracies.

We also reproduced the results both for MNIST and CIFAR-10 and used the last epoch accuracies as the reporting accuracies. The results are presented in Table 4a,b both for MNIST and CIFAR-10 datasets, respectively. The results show that our proposal achieves the highest accuracies of 94.95 ± 0.82 and 77.27 ± 0.58 for MNIST and CIFAR-10 datasets, respectively. Interestingly, the values of the compression rate *p* need to be adjusted properly due to the different behavior regarding how objects in the two datasets are represented. It requires at least 50% of the whole area of the image to fully isolate the main object in the MNIST images. In CIFAR-10, however, even when isolating 10% of the image area, the classifier can recognize the object in the image correctly. Figure 5 shows how the compression rate affects the output between the two datasets.

### 4.4. Limitations

The main bottleneck in our proposal is the additional computation costs to run the Cutout-CL process. For the MNIST and CIFAR-10 images, each sample is fed to the feedforward DNN 100 times to randomly place the inverse cutout windows to find the lowest loss samples. On average, it requires an additional 5 min to perform the process at the end of each task. Subsequently, performing this task with only 10 search times on the DAS data requires a much longer time. Our best-performing model requires around 11 h to perform such a process on (only) 2043 number of spectrogram samples. Additional limitation in our work relates to the fixed size window used for data isolation. Each object in every image often has a different size; therefore, using a fixed size window to isolate objects not only limits the type of object we can compress but also (for larger windows) it reduces the effective storage we can reallocate. For future work, we argue that developing an adaptive isolation, such as attention-based positional encoding, would not only solve the computation cost problem but could also addresses the optimum window size problem, making our proposal more favorable for the CL-based applications.

## 5. Conclusions

We presented the Cutout-CL, an iterative compressed continual learning pipeline based on the inverse cutout technique to isolate only the relevant parts of the input data and use them for rehearsal, increasing the available memory space in the process. In addition, we also proposed a simple and intuitive data augmentation method applicable to non-natural image datasets. Throughout extensive evaluation, we showed that our proposals provide significant improvements in term of higher $ACC_{T}$ and $BWT_{T}$ for modeling the DAS data by providing around 8% accuracy improvement compared to other CL algorithms, aiming to deliver a robust distributed virtual flow meter application.

Additionally, using the CL benchmark datasets, including MNIST and CIFAR-10 datasets, we showed that our proposal can also be applied to such datasets, achieving state-of-the-art accuracies. Using the the CIFAR-10 dataset on blurry10 and blurry30 settings, for example, our proposal provided the highest accuracies of 80.83% and 88.91%, respectively. Finally, using a relatively small *p*, we showed that the experimental evidence supports our hypothesis that by only storing relevant parts of the data and using them for retraining a model, we can acquire a better model than the model trained using the full size of input data.

## Figures and Tables

**Table 1:** Experiment results from offline and continual learning using DAS-Spectrogram dataset on different augmentation settings.

(**a**) Offline Learning Results in $ACC_{joint}$			
**n_cut = 1**	**n_cut = 5**	**n_cut = 20**	
40.83	46.45	48.08	
(**b**) CL Learning Results			
**Methods**	${\mathrm{ACC}}_{8}$	${\mathrm{BWT}}_{8}$	$I_{8}$
n_cut = 1	24.11	−40.66	16.72
n_cut = 5	25.44	−40.16	21.01
n_cut = 20	29.73	−39.51	18.35

**Table 2:** Spectrogram-DAS CL accuracy.

Methods	${\mathrm{ACC}}_{5}$	${\mathrm{BWT}}_{5}$	$I_{5}$
GDumb	18.20	0.31	29.88
RM	24.85	−40.50	23.23
RWalk	25.89	-	22.19
EWC	25.15	-	22.93
Cutout-CL			
No-Aug	27.22	−42.66	20.86
p=0.05	32.10	−28.05	15.98
p=0.10	29.73	−32.49	18.35
p=0.25	26.63	−42.90	21.45
p=0.50	25.44	−39.09	22.64

**Table 3:** $ACC_{5}$ of CL algorithms on CIFAR-10.

Methods	Disjoint	Blurry10	Blurry30
GDumb	42.47 ± 1.15	43.16 ± 0.77	45.72 ± 0.64
BiC	59.53 ± 4.30	61.45 ± 6.25	71.93 ± 2.45
RM	61.91 ± 0.63	76.86 ± 0.04	85.10 ± 0.16
RWalk	65.04 ± 0.11	78.59 ± 1.37	85.18 ± 0.57
iCaRL	65.61 ± 2.57	57.07 ± 2.74	64.90 ± 7.95
EWC	64.00 ± 1.34	78.67 ± 1.06	85.00 ± 0.42
Cutout-CL			
(p=0.1)	65.43 ± 2.83	80.83 ± 0.53	88.91 ± 0.20

**Table 4:** Last accuracies on (**a**) MNIST and (**b**) CIFAR-10 datasets.

(**a**) Results on MNIST Dataset		
**Methods**	${\mathrm{ACC}}_{5}$	$I_{5}$
GDumb	88.21 ± 0.63	10.03 ± 0.61
BiC	91.11 ± 0.13	7.13 ± 0.05
RM	94.35 ± 0.36	3.88 ± 0.34
EWC	89.51 ± 1.51	8.72 ± 1.60
Cutout-CL		
(p=0.25)	89.19 ± 0.51	9.05 ± 0.60
(p=0.50)	94.51 ± 0.97	3.73 ± 1.08
(p=0.75)	94.95 ± 0.82	3.29 ± 0.93
(**b**) Results on CIFAR-10 Dataset		
**Methods**	${\mathrm{ACC}}_{5}$	$I_{5}$
GDumb	44.87 ± 0.82	48.95 ± 1.04
BiC	60.89 ± 5.13	32.93 ± 4.94
RM	72.27 ± 1.31	21.55 ± 1.08
EWC	74.12 ± 1.51	19.70 ± 1.28
Cutout-CL		
(p=0.10)	77.27 ± 0.58	16.55 ± 0.45
(p=0.25)	75.08 ± 0.93	18.74 ± 0.70
(p=0.50)	73.91 ± 0.79	19.91 ± 0.67

## Data Availability

Not applicable.
